# Temperature of Chambers

**Published:** 1862-08

**Authors:** 


HALL'S JOURNAL OF HEALTH.

Vol. IX.] AUGUST. [No. 8.

### TEMPERATURE OF CHAMBERS.

Human life would be prolonged, and an incalculable amount of disease prevented, if a little fire were kept burning on the hearth during the night, winter and summer, if the doors and windows are kept closed. One great advantage would be, that a constant draft would be kept through the room, fireplace, and chimney, making a great degree of atmospherical vitiation impossible. There is a baleful error in the popular mind as to the nature and effects of pure air, warm air, and cold air. Warm air may be as pure as that of the poles; and although cold air is almost a synonym of pure air, and although it is healthful to breathe a cold air asleep or awake, yet the breathing of cold air is healthful only to a certain extent. It is not true that because it is healthful to sleep in a cool room, it is more healthful to sleep in a very cold room, not only because, as has been previously stated, carbonic acid becomes heavy under a great cold, and falls from the ceiling to the floor and bed of the sleeper, but because also a great degree of cold in a room where one is sleeping is very certain to cause dangerous and even fatal forms of congestion in the brain and lungs. The same ailments result from keeping sitting or sleeping apartments overheated. In midwinter, the heat of a sitting-room should not exceed sixty degrees of Fahrenheit, five feet above the floor. In the chambers of the sick in French hospitals, the directors are careful that there shall not be a greater heat than sixty degrees or about fifteen centigrade. The temperature of a sleeping apartment for invalids and for children in health should range about fifty degrees in cold weather, and not run lower than thirty-five; there is no advantage in sleeping in a colder atmosphere. Five hundred cubic inches of pure air should be delivered to invalids and sleepers every hour, as is the custom in the best-regulated French hospitals.

